# An extended set of yeast-based functional assays accurately identifies human disease mutations

**DOI:** 10.1101/gr.192526.115

**Published:** 2016-05

**Authors:** Song Sun, Fan Yang, Guihong Tan, Michael Costanzo, Rose Oughtred, Jodi Hirschman, Chandra L. Theesfeld, Pritpal Bansal, Nidhi Sahni, Song Yi, Analyn Yu, Tanya Tyagi, Cathy Tie, David E. Hill, Marc Vidal, Brenda J. Andrews, Charles Boone, Kara Dolinski, Frederick P. Roth

**Affiliations:** 1 Donnelly Centre, University of Toronto, Toronto, Ontario M5S 3E1, Canada;; 2 Department of Molecular Genetics, University of Toronto, Toronto, Ontario M5S 3E1, Canada;; 3 Department of Computer Science, University of Toronto, Toronto, Ontario M5S 3E1, Canada;; 4 Lunenfeld-Tanenbaum Research Institute, Mt. Sinai Hospital, Toronto, Ontario M5G 1X5, Canada;; 5 Department of Medical Biochemistry and Microbiology, Uppsala University, SE-75123 Uppsala, Sweden;; 6 Lewis-Sigler Institute for Integrative Genomics, Princeton University, Princeton, New Jersey 08544, USA;; 7 Center for Cancer Systems Biology (CCSB), Dana-Farber Cancer Institute, Boston, Massachusetts 02215, USA;; 8 Department of Genetics, Harvard Medical School, Boston, Massachusetts 02115, USA;; 9 Canadian Institute for Advanced Research, Toronto, Ontario, M5G 1Z8, Canada

## Abstract

We can now routinely identify coding variants within individual human genomes. A pressing challenge is to determine which variants disrupt the function of disease-associated genes. Both experimental and computational methods exist to predict pathogenicity of human genetic variation. However, a systematic performance comparison between them has been lacking. Therefore, we developed and exploited a panel of 26 yeast-based functional complementation assays to measure the impact of 179 variants (101 disease- and 78 non-disease-associated variants) from 22 human disease genes. Using the resulting reference standard, we show that experimental functional assays in a 1-billion-year diverged model organism can identify pathogenic alleles with significantly higher precision and specificity than current computational methods.

Rapidly evolving high-throughput sequencing technology has enabled routine identification of both rare and common sequence variants within individual humans. There is now an urgent need to identify those genetic variations with the greatest potential to impact human health. Genetic linkage and association studies have connected tens of thousands of genetic loci with diverse human diseases, but the disease-causing variants have been identified for only a small fraction of these loci (Stranger et al. 2011). Even for genes that have been unambiguously identified as disease causing, the common variants tend to have small effects on disease risk. The 100–400 rare missense coding variants carried by each human are likely to have the greatest impact on health and disease (Pritchard 2001; Kryukov et al. 2007; Marth et al. 2011; Nelson et al. 2012; Tennessen et al. 2012). Unfortunately, the rarity of these variants limits the power to detect correlation of these variants with disease, so that they are typically not found in genome-wide association studies. Improved identification of the subset of germline or somatic mutations that are pathogenic can enable disease risk assessment for individual patients and, with increasing frequency, improve therapy (Domchek et al. 2013; Burgess 2015). Moreover, improved interpretation of missense variants is needed for carrier screening and pre-implantation embryonic screening (Bell et al. 2011; Francescatto and Katsanis 2015).

Additional applications of pathogenic mutation detection can be anticipated. Association between a gene and a disease can be established by comparing the burden of rare variants in human disease cases relative to controls (Purcell et al. 2014). The use of ‘deleteriousness’-weighted mutational counts can improve the power of burden-of-mutation studies, further highlighting the importance of better technology for identifying deleterious variants. Application of clustered regularly interspaced short palindromic repeat (CRISPR) technology has enabled (for better or for worse) (Baltimore et al. 2015; Bosley et al. 2015) germline editing as well as regenerative medicine based on ex vivo engineering of induced pluripotent stem cells (iPSCs) and reintroduction of differentiated cells; however, the functionality of off-target and other passenger mutations still needs to be rigorously examined for safety (Prowse et al. 2014; Baltimore et al. 2015).

Diverse computational and experimental methods exist to infer the pathogenicity of rare human coding variants. Although computational methods are beginning to find acceptance as clinical diagnostic tools (Richards et al. 2015), they have limited predictive power (Mathe et al. 2006; Chan et al. 2007; Cline and Karchin 2011; Thusberg et al. 2011; Castellana and Mazza 2013; Frousios et al. 2013; Gnad et al. 2013). Experimental assessment of variant function in human cells is hampered by inefficient allele replacement technology and by the presence of paralogs with overlapping functions, making complementation testing in ‘humanized’ model organisms an attractive alternative (Marini et al. 2008; Trevisson et al. 2009; Mayfield et al. 2012; Davis et al. 2014; Laurent et al. 2015). Complementation of mutant versions of model organism genes by cognate human genes is a classic method to identify human gene function (Lee and Nurse 1987; Levitan et al. 1996; Osborn and Miller 2007; Keegan et al. 2011). Such complementation relationships can then be exploited to assess the impact of amino acid changes in human proteins (Kruger and Cox 1994, 1995; Marini et al. 2008; Trevisson et al. 2009; Wei et al. 2010; Mayfield et al. 2012; Dimster-Denk et al. 2013; Davis et al. 2014). Like computational methods, complementation assays in experimentally tractable model organisms allow functional assessment of human variation at a scale that is commensurate with the size of the human population. However, a rigorous comparison of the performance of computational and experimental methods has not been available.

To compare the performance of experimental and computational methods in identifying pathogenic human variants, we developed a set of complementation assays that represents a surrogate genetics platform for identifying pathogenic human variants. We evaluated this platform by constructing expression vectors for both disease- and nondisease variants across 22 human disease genes. This allowed us to assess how well failure of a variant clone to complement could predict variant pathogenicity and to compare predictive success with that of current computational methods.

## Results

### Developing a reference set of yeast/human functional complementation relationships

**Figure 1.**
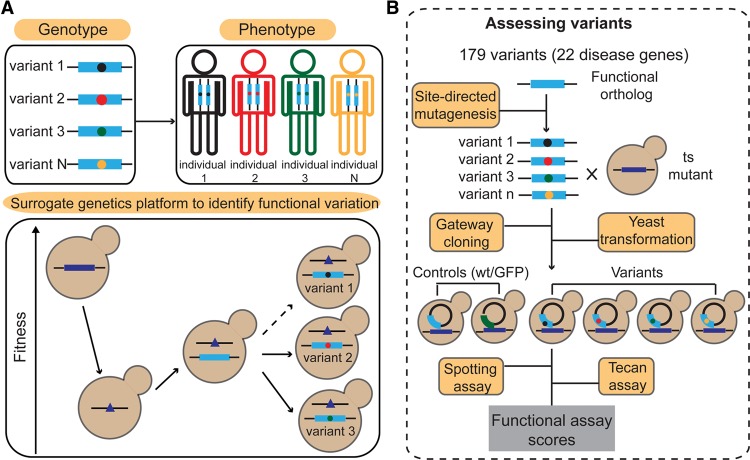
Overview of the surrogate genetics platform for functional assessment of genetic variants. (*A*) Humanized yeast as a surrogate genetic system to evaluate functional effects of human genetic variation. (1) Disruption of a yeast gene gives rise to a yeast phenotype (e.g., decreased fitness). (2) The yeast phenotype is rescued by wild-type human alleles. (3) Functional effects of human variants are evaluated based on their ability to rescue the phenotype relative to the wild-type allele. A human variant may be deemed pathogenic if it cannot rescue the phenotype as well as wild type, or in special cases, where it exhibits better-than-wild-type rescue of the phenotype. (*B*) Overview of functional assessment of variants in disease-associated genes.

To assess the ability of “surrogate genetics” (Kruger and Cox 1995; Marini et al. 2008; Mayfield et al. 2012) to reveal functionality of human disease gene variants, we developed a reference set of yeast-based functional complementation relationships for human genetic variants. Functional complementation assays in yeast can be developed via two basic steps (Fig. 1A): (1) Complementation relationships are identified in which a human gene rescues phenotypic defects of a loss-of-function mutation in a cognate yeast gene; and (2) the pathogenicity of each genetic variant is assessed by measuring its ability to complement.

To develop a reference set of complementation relationships, we tested the ability of 139 ‘wild-type’ human disease genes to rescue temperature-sensitive mutations in 125 orthologous yeast essential genes (Supplemental Fig. S1A; Supplemental Table S1; Supplemental Note). Each ‘wild-type’ human disease gene, expressed via a constitutive promoter on a low-copy plasmid, was transformed into the corresponding yeast temperature-sensitive (ts) strain in parallel with a negative control plasmid expressing green fluorescent protein (GFP) (Supplemental Fig. S1B; Methods). A complementation relationship was identified when the human ortholog rescued growth of the yeast ts strain at a nonpermissive temperature (Supplemental Fig. S2A). Of the 139 orthologous pairs tested, 26 complementation relationships were identified (Supplemental Fig. S3; Supplemental Table S1). To compare these relationships with previous knowledge, we carried out extensive literature curation of complementation relationships between human and yeast (Supplemental Table S2; Supplemental Fig. S2B; Supplemental Note). Among the gene pairs we tested, there were 12 previously reported complementation relationships, of which seven (58%) were recapitulated in our data set. Of the 26 complementation relationships we observed, 19 (73%) were novel. For a subset of 19 complementing pairs for which a heterozygous mutant of the yeast gene was available (Supplemental Note), we further tested whether the human gene could rescue a yeast null mutant and confirmed complementation in all cases (Supplemental Fig. S4). Thus, we established functional assays for 26 human disease genes, of which 19 were novel.

### Sequence features cannot confidently predict complementing pairs

We wondered whether complementation relationships could be confidently predicted from sequence-based characteristics. We found that sequence identity was higher for complementing pairs (45%) than noncomplementing pairs (29%) (*P* = 0.0006; Wilcoxon test) but not a perfect predictor of functional rescue (Supplemental Fig. S2C). The fraction of yeast residues aligned with the human gene had only a suggestive but not a significant correlation with complementation (Wilcoxon test, *P* = 0.09) (Supplemental Fig. S2D). For complementing pairs, it was slightly more likely for all protein domains in the yeast protein to be present in the human counterpart (Fisher's exact test, *P* = 0.018) (Supplemental Fig. S2E,F). No statistical differences were seen in a similar analysis of literature-curated complementing and noncomplementing yeast-human orthologous pairs (Supplemental Table S2; Supplemental Fig. S2G–I). Together, these results suggest that sequence features alone cannot confidently predict whether a human gene will complement its yeast counterpart.

### Assessing the pathogenicity of missense variants

Having established a reference set of yeast/human functional complementation relationships, we next exploited these functional assays to evaluate the cross-species complementation strategy for predicting human variant pathogenicity. Throughout the text, we use “disease-associated” to indicate that there is some evidence (strong or weak) to associate a variant with diseases and “non-disease-associated” to indicate variants that have not been annotated as being associated with any disease. Disease-associated missense variants were known for 22 of the 26 human disease-associated genes for which complementation relationships were identified. For these 22 genes, we constructed and sequence-confirmed clones for 101 disease- and 78 non-disease-associated variants (see Supplemental Note for details). Missense variants were constructed by site-directed mutagenesis (see Methods), transferred to a yeast expression plasmid by Gateway technology, and individually transformed into the corresponding yeast ts mutant strains (Fig. 1B).

**Figure 2.**
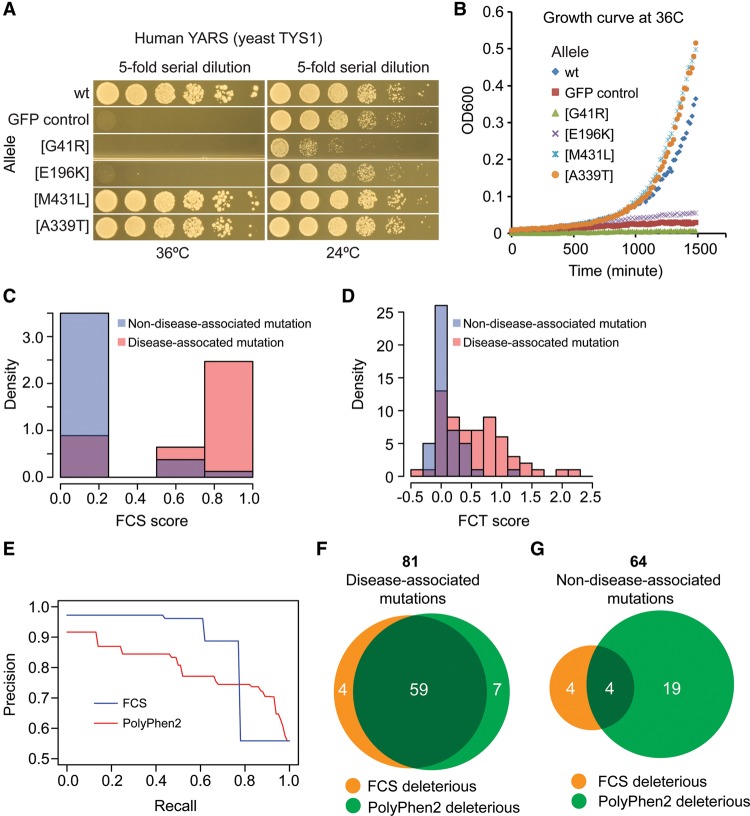
Assessment of functional effect of missense variants. (*A*,*B*) Growth assays on solid media (*A*) and liquid media (*B*) for human tyrosyl-tRNA synthetase (*YARS*) gene variants. G41R and E196K are disease-associated variants; M431L and A339T are non-disease-associated variants. The yeast cells were temperature-sensitive mutants of the yeast *TYS1* gene, expressing either wild-type or variant alleles of the *YARS* gene, or the *GFP* gene as a control. For solid growth assays, fivefold serial dilutions of yeast cells were spotted onto plates and incubated at 24°C and 36°C for 2 d. For liquid growth assays, approximately 10^5^ cells were inoculated in 100 μL liquid medium and the absorbance at 600 nm was read every 15 min. (*C*) Distribution of solid growth (FCS) scores for disease- and non-disease-associated variants. (*D*) Distribution of liquid growth (FCT) scores for disease- and non-disease-associated variants. (*E*) Precision and recall analysis for FCS and PolyPhen2 scores. (*F*) Overlap of disease-associated variants predicted to be deleterious by functional assays (orange) and PolyPhen2 (green). (*G*) Overlap of non-disease-associated variants predicted to be deleterious by functional assays (orange) and PolyPhen2 (green). As described in the text, analyses in this figure exclude FCT assays of *PKLR* variants and all assays of *UROS* variants.

To assess the functionality of each of these 179 variants, we assessed the growth of serially diluted “spotted” cells on solid media and via spectrophotometric time courses in liquid media (Fig. 2A,B). Each variant was assayed alongside negative and positive controls for loss of complementation (expression of either the wild-type human protein or a GFP control). Two functional scores—the functional complementation by spotting (FCS) score and functional complementation by liquid growth time-course (FCT) score—were assigned as described in Methods (Supplemental Fig. S5; Supplemental Tables S3, S4). To enable a comparison with computational methods for predicting the functional impact of missense variants, scores were obtained from a widely used computational method, PolyPhen2 (Supplemental Table S3; Adzhubei et al. 2010). All 34 variants in the uroporphyrinogen III synthase gene (Hs*UROS*; the prefixes “Sc” and “Hs” are used throughout the text to indicate *S. cerevisiae* or *H. sapiens*, respectively) were excluded because, in contrast with all other genes examined, these variants tended to exhibit faster-than-wild-type growth (Supplemental Figs. S5, S6). Functional effects of the Hs*UROS* variants were further assessed separately. In total, scores were assigned for 145 variants.

Correlation was high between replicate measures for the semiquantitative FCS score based on spotting assays (PCC = 100%) and between replicates of relative growth rates in liquid media (PCC = 91%) (Supplemental Fig. S7A,B). Correlation between FCS and FCT scores was also high (PCC = 81%), suggesting that complementation is robust in different assays (Supplemental Fig. S8A). Although growth assays in liquid media are more quantitative than spotting assays on solid media, the growth defect of some yeast ts mutants manifested more clearly in the solid growth assay. For example, the wild-type human pyruvate kinase (Hs*PKLR*) gene was observed to complement the yeast pyruvate kinase (Sc*CDC19*) ts mutant only using solid and not liquid growth assays (Supplemental Fig. S5; Supplemental Table S4), so that variants of PKLR were excluded from FCT score analysis.

### Evaluating the performance of pathogenicity predictions

Distributions of complementation scores, plotted separately for disease- and non-disease-associated variants (Fig. 2C,D), clearly show that both FCS and FCT functional complementation assays can separate disease- from non-disease-associated variants. Both FCS and FCT exhibited correlation (PCC = 57% and 45%, respectively) with the previously validated PolyPhen2 method (Supplemental Fig. S8B,C; Adzhubei et al. 2010), further supporting the ability of complementation assays to detect pathogenic variants.

Using the subset of 61 disease- and 45 non-disease-annotated variants with both FCS and FCT scores, we evaluated precision (fraction of disease-variant predictions that were correct according to our reference set) and recall (fraction of all annotated disease variants that were predicted) and found FCS and FCT to perform similarly (Supplemental Fig. S9A). Therefore, we proceeded only with the FCS score because it was available for more variants (81 disease- and 64 non-disease-associated). We next compared FCS with PolyPhen2, a widely used computational method for predicting deleterious variants. The precision-recall curve (Fig. 2E) shows that yeast-based functional complementation assays outperform PolyPhen2 in terms of pathogenicity prediction. The same result held when we expanded this analysis to three additional computational methods, SIFT (Ng and Henikoff 2001), PROVEAN (Choi et al. 2012), and CADD (Supplemental Fig. S9B; Kircher et al. 2014).

As an example performance point, a threshold value of 0.6 for the FCS score achieves a precision of 89% and recall of 78% (Fig. 2E). At a PolyPhen2 score threshold (0.56), which matches this recall performance, the precision of FCS scoring (89%) significantly exceeds that of PolyPhen2 (precision 74%; Fisher's exact test, *P* = 0.008). At this recall level (78%), the specificity of FCS scoring (88%) also significantly exceeds that of PolyPhen2 (specificity 64%; Fisher's exact test, *P* = 0.001). Analysis of overlap between experimental and computational predictions (Fig. 2F) shows that they largely predict the same disease variants to be deleterious. Thus, the improved performance of complementation testing is best understood in terms of its heightened specificity, i.e., its ability to correctly identify nonpathogenic variants: Of the 64 non-disease-associated variants, PolyPhen2 predicted 23 (36%) of these non-disease variants to be deleterious, as compared with only eight (13%) for the FCS score (Fisher's exact test, *P* = 0.001) (Fig. 2G).

We next examined whether prediction performance could be improved by combining FCS and PolyPhen2 scores. To achieve a common scale, precision-calibrated versions of FCS and PolyPhen2 scores (FCS′ and PolyPhen2′) were generated (see Methods). Precision and recall performance was then evaluated for seven methods of combining the two calibrated scores: minimum, maximum, mean, and four alternative weighted means (w1 through w4; see Methods) (Supplemental Fig. S9C,D). The results show that combining FCS and PolyPhen2 scores can improve the performance in the high precision/low recall region and can permit ranking among the many ties that result from using only the semiquantitative FCS or FCS′ scores.

We further calculated several single-point performance estimates: (1) area under the precision-recall curve (AUPRC) and (2) Matthews correlation coefficient (MCC), each representing a different way of combining precision and recall performance; (3) area under the receiver-operating characteristic curve (AUROC), which estimates the probability that the prediction method will properly rank-order two randomly chosen mutations (one pathogenic and the other neutral); and (4) recall at 90% precision (REC90), which measures the fraction of pathogenic mutations that can be confidently predicted (Table 1). Although the precise level of precision required by a clinician to make a diagnostic or therapeutic decision necessarily depends on many factors, it seems clear that any clinical decision should require a high level of precision. We therefore argue that recall at a high precision (exemplified here by REC90) is the most clinically relevant measure of performance.

**Table 1.**
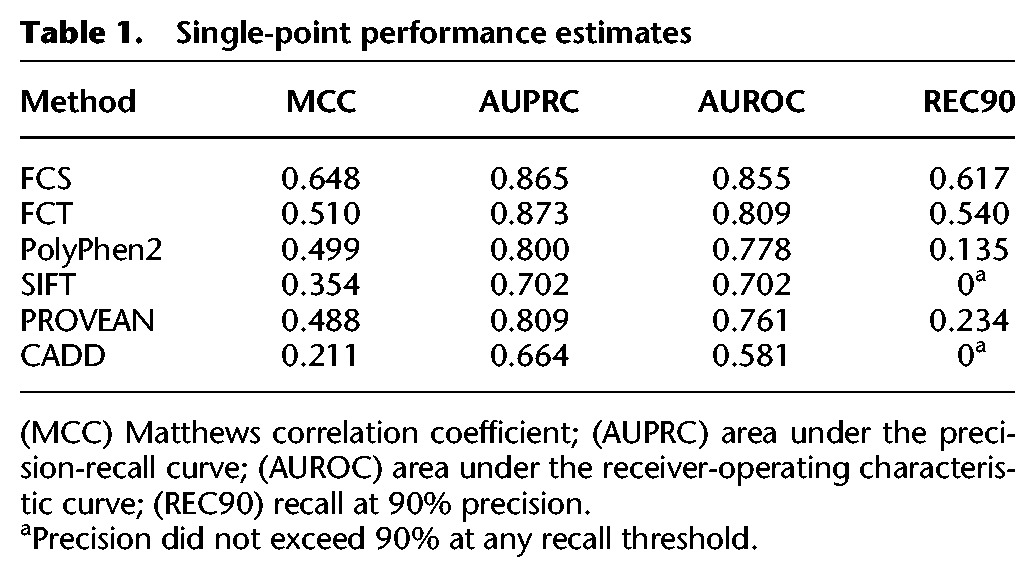
Single-point performance estimates

For each of these performance measures, functional assays substantially outperformed every computational method examined, with the performance difference being most dramatic for the most clinically relevant REC90 measure. Among the computational methods, PROVEAN had the best performance for the REC90 measurement at 23%, as compared with 62% for the complementation-based FCS score.

### Pathogenicity was systematically less well predicted for one gene—Hs*DPAGT1*

We systematically sought genes for which pathogenicity was predicted unusually well or poorly. Pathogenic mutations for only one gene—Hs*DPAGT1*—were predicted significantly less well than were other genes, both using complementation-based FCS score (Fisher's exact test, *P* = 0.0002) and also using PolyPhen2 (Fisher's exact test, *P* = 0.005). We could find no clear explanation for this, but note that the pathogenic mutations we examined for Hs*DPAGT1* mutations had a suggestive (but nonsignificant) tendency to appear at positions that were less conserved between yeast and human proteins than the other mutations we examined (Wilcoxon test, *P* = 0.09).

### Evaluating Hs*UROS* variants

As noted above, the Hs*UROS* gene (complementing mutations in Sc*HEM4*) differed from other genes in that its variants tended to exhibit faster growth than yeast cells carrying a wild-type Hs*UROS* gene (Supplemental Figs. S5, S6). The fact that each of the disease-associated Hs*UROS* variants we tested are recessive loss-of-function mutations (Shady et al. 2002) is consistent with a scenario in which the wild-type Hs*UROS* gene mimics a state of overexpression or hyperactivation of the orthologous counterpart Sc*HEM4* gene, thus leading to a growth defect. Under this model, the disease-causing Hs*UROS* variants alleviate this growth defect by decreasing enzymatic activity and/or expression, while still maintaining an activity that is sufficient to support growth. This model is supported by two previous studies: (1) a systematic study showing that overexpression of the Sc*HEM4* gene reduces growth rate (Sopko et al. 2006); and (2) a study suggesting that the azaoxoaporphine alkaloid sampangine inhibits heme synthesis by hyperactivating the Sc*HEM4* gene (Huang et al. 2011). The latter study showed that either sampagine treatment or Sc*HEM4* overexpression causes a growth defect that is remediable by heme supplementation. Although the 34 Hs*UROS* variants were eliminated from the analysis above, we calculated an alternative ‘reversed’ variant of the FCS and FCT score (Methods). Interestingly, distributions of the reversed FCS and FCT scores for Hs*UROS* variants (Supplemental Fig. S10) clearly show separation of disease- and non-disease-associated variants by both functional complementation assays. Precision-recall results were similar with (Supplemental Fig. S9E,F) and without (Supplemental Fig. S9A,B) the alternatively scored Hs*UROS* variants, so that Hs*UROS* variants were included in subsequent analyses.

### Complementation assays may be identifying errors in pathogenicity annotation

Apparent errors in variant pathogenicity predictions might in fact correspond to errors in the literature or its annotation, i.e., occurring due to ‘contamination’ of our reference variant set. Such contaminations will tend to make the prediction problem artificially more difficult, so that performance estimates will tend to be conservative underestimates.

**Figure 3.**
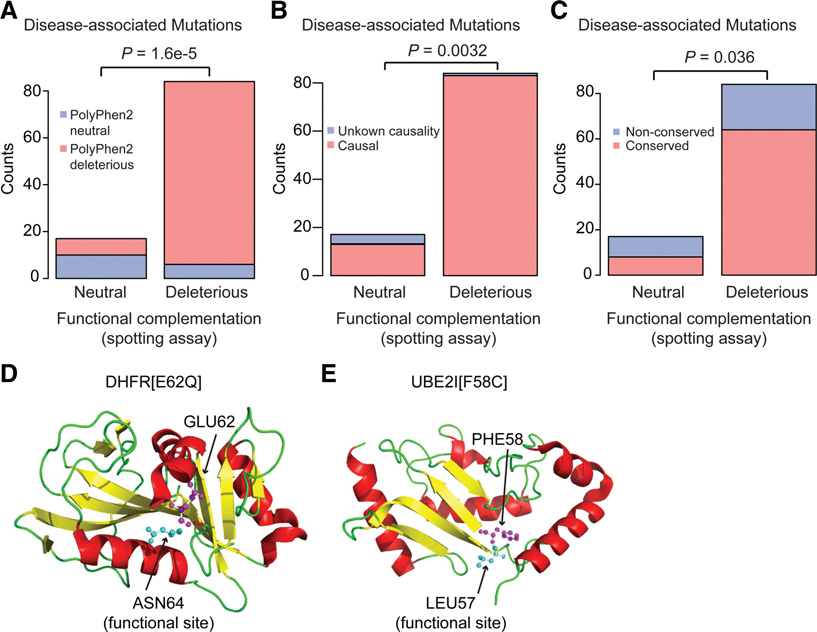
Exploring differences between functional assays and current pathogenicity annotation. (*A*–*C*) Among variants currently annotated as disease-associated, those classified as neutral by the FCS assay (apparent false negatives) overlap significantly with (*A*) variants that are also classified as neutral by PolyPhen2 (*P* = 1.6 × 10^−5^, Fisher's exact test); (*B*) disease-associated variants with unknown causality (*P* = 0.0032, Fisher's exact test); and (*C*) variants located at nonconserved residue positions. Enrichment in each case was relative to the corresponding frequency among variants called deleterious by the FCS assay (*P* = 0.036, Fisher's exact test). (*D*,*E*) Among non-disease-associated variants classified as deleterious by functional assays (apparent false positives), there were two variants for which structural context supported the finding of these variants to be deleterious: (*D*) DHFR[E62Q] and (*E*) UBE2I[F58C]. (Pink) Residues where variants occur; (cyan) functional sites.

We first explored the possibility that some of the variants annotated as pathogenic but missed by the complementation assay might in fact be nonpathogenic. Of the 101 disease variants we tested, experimental functional assays predicted 83 (82%) to be deleterious at the 0.6 FCS threshold described above. Of the 78 not-annotated-as-disease-associated (‘nondisease’) variants we tested, 68 (87%) were predicted neutral. Among the 101 disease-associated variants, 18 were classified as neutral by the FCS score. In keeping with the idea that apparent errors of the FCS assay might in fact correspond to annotation errors, FCS-neutral ‘errors’ were enriched (relative to the FCS-deleterious ‘correct’ predictions) for variants classified as PolyPhen2 neutral (using the matched-recall threshold of 0.56; Fisher's exact test, *P* = 1.6 × 10^−5^). Such ‘errors’ were also enriched among HGMD variants annotated for disease-associated but not disease-causal annotations (Fisher's exact test, *P* = 0.003) (Fig. 3A,B). Furthermore, where FCS made neutral predictions that were errors according to our reference set, these were enriched for variants in nonconserved residue positions (Fisher's exact test, *P* = 0.036) (Fig. 3C; Supplemental Table S3). Taken together, these analyses suggest that at least some of the reference disease variants predicted by FCS to be nonpathogenic are in fact nonpathogenic despite annotations as disease mutations in HGMD.

We next explored the possibility that some of the ‘false positives’ of the complementation assay might in fact be pathogenic variants. Among the 78 non-disease-associated variants examined, 10 variants in seven unique genes showed complete or partial loss of complementation in functional assays. We examined the structural context of two such variants for which a protein crystal structure was available: UBE2I[F58C] and DHFR[E62Q]. Both residues are in close proximity to functional sites (pairwise distances of 3.8 Å between alpha-carbons of PHE58 and functional site residue LEU57 for the gene Hs*UBE2I* and 5.2 Å between GLU62 and functional site residue ASN64 for the gene Hs*DHFR*), making it plausible that these two variants do in fact alter the functions of their proteins (Fig. 3D,E).

### Potential impact of annotation errors and allele frequency on performance estimates

To examine the sensitivity of our main conclusion to our choice of positive reference set, we generated three more confident reference sets by (1) removing five variants that are not annotated as DM (“disease causing”) in HGMD from the disease-mutation reference set, (2) examining the intersection between HGMD (mutations annotated as DM) and another mutation database ClinVar (Landrum et al. 2014) (considering only those mutations annotated in ClinVar as pathogenic), and (3) examining mutations found only in the HGMD disease-causing ‘DM’ set but not in ClinVar (Supplemental Table S3). The performance of complementation assays relative to computational predictions increased as more stringent filtering was applied to the disease set: performance ratio of FCS relative to PolyPhen2 in terms of AUPRC was 1.08 for the original reference set (Supplemental Fig. S11A), 1.09 for the HGMD DM-restricted reference set (Supplemental Fig. S11B), 1.14 for the intersection of HGMD DM and ClinVar pathogenic (Supplemental Fig. S11C), and 1.24 for mutations found only in the HGMD DM set (Supplemental Fig. S11D). Thus, for each alternative choice of positive reference set, complementation assays outperformed PolyPhen2.

To examine the potential impact on prediction performance from false positives in HGMD, which have been previously estimated to occur at a rate as high as 27% (Bell et al. 2011), we simulated the impact of randomly adding in benign mutations such that the positive reference set consists of 73% stringently annotated disease mutations (drawn from the intersection of HGMD-DM and ClinVar pathogenic mutations) and 27% nondisease mutations randomly selected from the negative reference set. To account for the effect that changing the prior probability of disease mutations can have on precision estimates, performance was estimated using the ratio of AUPRC relative to the prior probability (designated as AUPRC_norm) instead of AUPRC. The AUPRC_norm was calculated for 10,000 simulated reference sets and plotted together with the corresponding AUPRC_norm for the ‘uncontaminated’ stringent reference set shown in red dots (Supplemental Fig. S12). The results showed that the false annotation of benign alleles in the reference set would cause underestimates of prediction performance (the median AUPRC_norm of simulated reference sets was 1.42 for FCS and 1.37 for PolyPhen2, while the AUPRC_norm of the original reference set was 1.99 for FCS and 1.76 for PolyPhen2). Thus, our performance estimates are conservative.

To directly assess whether annotation errors were abundant in our positive reference set, we recurated the literature for a subset of 20 mutations. These were selected uniformly at random from the 96 HGMD-DM mutations in our positive reference set. Following the ClinVar curation standard (Richards et al. 2015), we found two mutations (10%) to have uncertain causality (Supplemental Table S3).

We next investigated the performance as a function of allele frequency; we generated four new reference sets, each restricting to the intersection of HGMD DM and ClinVar pathogenic variants as described above but filtered differently based on allele frequencies available in the Exome Aggregation Consortium (ExAC) (Supplemental Table S3; Exome Aggregation Consortium et al. 2015). The four reference sets correspond to (1) ExAC, variants for which MAF information is available in ExAC, (2) ExAC1e-5, variants whose MAF is less than 0.00001, (3) ExAC1e-4, variants whose MAF is less than 0.0001, and (4) ExAC1e-3, variants whose MAF is less than 0.001. The precision and recall curves were plotted for the four new reference data sets (Supplemental Fig. S11E–H). Under all four different ExAC frequency thresholds, FCS performed better than PolyPhen2.

Although yeast complementation assays are more likely to miss the functional impact of dominant negative and gain-of-function alleles (Herskowitz 1987; van Oijen and Slootweg 2000), we note that the reference set of variants was not in any way selected on the basis of knowledge of these allele types, so that our performance estimates should be broadly representative.

Computational methods for predicting variant pathogenicity generally require a training set of pathogenic and nonpathogenic variants. It is therefore possible, if a computational method was trained with variants in our reference set, that the computational method has effectively ‘peeked’ at the test set. If overfitting has occurred, this could result in an artificially inflated performance estimate for the computational method. We examined this possibility for PROVEAN and PolyPhen2, the two computational methods that arguably performed best according to our reference set. This was not a relevant issue for PROVEAN, because it does not use a training set to rank variants by pathogenicity. For PolyPhen2, however, there was overlap between its training set and our reference set. After removing overlapping variants from our test set, performance of PolyPhen2 diminished slightly, so that the relative AUPRC performance of complementation increased from 1.08 to 1.16.

## Discussion

Here we systematically explored the idea of using yeast as a surrogate genetic system to identify functional human gene variation. For 26 human disease genes, we developed yeast-based functional assays to evaluate the functional effect of disease-associated mutations and non-disease-associated mutations. Of these 26, 19 (73%) represent novel complementation relationships.

At the outset of this study, it was not at all clear that yeast complementation assays would perform well as a platform for identifying pathogenic mutations, given the billion-year divergence between yeast and human cells. However, we were motivated by known limitations of computational methods (Marini et al. 2010). Empirically, using a ‘gold standard’ set of disease and nondisease mutations, we showed quantitatively that yeast complementation assays predict variant pathogenicity with high recall and specificity. Of the 101 disease alleles we tested, the assay showed 83 (82%) to be deleterious. Of the 78 alleles we tested that are not known to be associated with disease, 68 (87%) were neutral according to functional assays. As a pathogenicity assay, functional complementation outperformed all computational methods examined.

Functional assays have the potential to provide insight into disease severity, in that complementation by disease variants can be assessed quantitatively. Although the number of known disease variants with known and clearly characterized severity is small, we note two potential examples: In the first, two disease variants were tested for the Hs*GDI1* gene (Sc*GDI1*, yeast orthologous gene). Of these, L92P showed partial loss of complementation while R423P showed complete loss of complementation. The patient carrying the GDI1[L92P] variant had a relatively milder clinical phenotype compared to the patient carrying the GDI[R423] variant (Bienvenu et al. 1998; D'Adamo et al. 1998). In the second potential example, two disease variants, D268N and R206P, were assessed for the phosphoglycerate kinase 1 (Hs*PGK1*) gene (Sc*PGK1*, yeast orthologous gene), variants of which can cause phosphoglycerate kinase 1 deficiency. Both variants showed wild-type-like complementation in the functional assays, corresponding to reports of a mild reduction in biochemical activity and no overt clinical symptoms for D268N, and only mild clinical symptoms for R206P (Fujii et al. 1980; Fujii and Yoshida 1980).

Genetic association and sequencing technologies have enabled the discovery of associations between over 200 human traits and thousands of SNPs that represent human haplotypes (‘tagSNPs’) (Welter et al. 2014). However, distinguishing association from causality remains a critical unmet need. Previously proposed principles for establishing disease causality of human variants using model organisms (Chakravarti et al. 2013) require that four postulates be satisfied for a claim of causality: “(1) Candidate gene variants are enriched in patients. (2) Disruption of the gene in a model system gives rise to a model phenotype that is accepted as relevant and ‘equivalent’ to the human phenotype. (3) The model phenotype can be rescued with the wild-type human alleles. (4) The model phenotype cannot be rescued with the mutant human alleles.”

One variant that was disease associated but of unknown causality was the thiamine pyrophosphokinase 1 (Hs*TPK1*) variant G223R. This variant was previously identified as a de novo mutation in an autism patient (Sanders et al. 2012) and could thus be taken to satisfy Postulate 1. It is not entirely clear how one should decide whether Postulate 2 (that the yeast phenotype is accepted as relevant and “equivalent” to the human phenotype) is satisfied. However, the overall success of complementation-based prediction suggests satisfaction of Postulate 2. Interestingly, the G223R variant showed impaired complementation in both FCT and FCS assays, providing direct evidence that the G223R mutation makes a dysfunctional protein. Thus, complementation assays satisfied the remaining Postulates 3 and 4, establishing disease causality for the G223R variant according to the standard of Chakravarti et al. (2013).

We also identified new evidence for disease causality through analysis of the set of “nondisease” control alleles. A non-disease-associated variant of the GDP dissociation inhibitor 1 (Hs*GDI1*) gene (D256Y), a causal gene for one form of X-linked mental retardation (Bienvenu et al. 1998), showed partial loss of complementation. Upon further examination of the literature, we found that this variant is a common polymorphism that has been associated with intelligence levels of children in the Qinba region in China (Zhang et al. 2008), potentially satisfying Postulate 1. Together with the known connection of GDI1 to mental retardation, our results argue for satisfaction of the remaining causality postulates and for a closer investigation of this variant. Given the power of model organism-based assays to assess variant pathogenicity, it is worth asking whether this can be a general solution. Although we limited this study to human disease genes with an essential yeast ortholog, human disease genes with nonessential yeast orthologs can exhibit strong phenotypes in the right genetic background (Costanzo et al. 2010) or growth environment (Hillenmeyer et al. 2008). Given the rate at which we observed complementation (26 out of 139 or 19%) and our screen's apparent false negative rate (eight out of 15 or 53%), a yeast complementation assay might be expected for ∼40% of those human disease-associated genes with a yeast ortholog. This is consistent with a very recent study (Kachroo et al. 2015), for which 47% of one-to-one human/yeast ortholog pairs showed complementation. Extrapolating to the set of 1047 human disease-associated genes with a yeast ortholog, complementation assays are within reach for ∼500 disease-associated genes, covering 10% of the ∼5000 disease-associated genes annotated in OMIM or HGMD. This fraction will of course be much higher when all other tractable model organisms are considered, e.g., zebrafish as a model for cardiac developmental disorders (Musso et al. 2014).

Model organisms can provide a platform for functional analysis even in the absence of orthology. Examples include yeast-based genetic assays for interaction between two human proteins or between a protein and DNA. In one study, variants of the Hs*ABCB1* gene, encoding the major efflux pump (also called P-glycoprotein), were functionally evaluated in yeast (Jeong et al. 2007). In another study, mutagenesis of the human tumor suppressor gene Hs*TP53* was followed by the reverse yeast one-hybrid assay to identify variants that lose DNA-binding activity (Brachmann et al. 1996). Nearly all of the variants identified by this approach had also been observed to be somatically mutated in cancer patient samples (Brachmann et al. 1996).

Surrogate genetics in humanized model organisms offers additional benefits. In yeast and several other model organisms, it is possible to efficiently identify genetic modifiers at a genome scale (Lehner et al. 2006; Byrne et al. 2007; Costanzo et al. 2010; Horn et al. 2011). Modifier screens in model organisms offer a powerful means not only for understanding disease mechanism and identifying additional disease genes, but also for identifying potential therapeutics (Tardiff et al. 2014). The experimental throughput of model organisms offers the opportunity to build rich genotype-phenotype maps through saturation mutagenesis and deep sequencing—see, for example, Fowler et al. (2014) and Kitzman et al. (2015)—potentially providing a ‘look-up table’ for functionality of human variants before they have ever been observed in the clinic.

Ideally, the functional effect of human genetic variants would be evaluated in human cells. Unfortunately, it can be challenging to identify a phenotype in human cells that is strong enough to quantitatively evaluate functional variation. Based on a set of high-performing shRNA screens across different human cancer cell lines (Marcotte et al. 2012), Hart et al. compiled a list of 291 genes that showed some growth phenotype in at least half of 48 cell lines (Hart et al. 2014). Only one (4%) of the 26 complementing human disease genes identified in this study yielded a fitness effect in Hart et al. Even accepting cases where the phenotype was observed in only a single cell line within either of two large-scale shRNA screens (Cheung et al. 2011; Marcotte et al. 2012) (and assuming that all of these phenotypes are strong enough to form the basis of a robust functional assay), only 13 (50%) of the 26 complementing human genes in this study showed any shRNA phenotype.

How and whether predicting pathogenic mutations from either complementation or computational studies will be useful is context dependent. Where the prior probability of a pathogenic mutation is low—for example, in asymptomatic patients without a family history of disease or when performing naive carrier screening—predictions of pathogenicity should be viewed more skeptically. Where a patient presents with a particular disease, however, there is a higher prior probability of a causal mutation in genes associated with that disease, so that pathogenic mutations in the relevant genes can be more confidently identified. The results of complementation assays can be used in different settings with different prior probabilities, e.g., by inserting estimates of prior probability and likelihood ratio from the functional complementation assay (Supplemental Table S6) into the odds form of Bayes’ Rule (Supplemental Note).

Computational predictions of deleterious mutation are already used to weigh the deleteriousness of mutations in the burden-of-mutation studies, reducing the ‘noise’ of neutral mutations that are less likely to be related to the trait (Carter et al. 2013). Complementation-based identification of deleterious mutation, where possible, could further improve burden-of-mutation studies.

Complex genetic diseases, where a mutation is pathogenic only in the context of other mutations, represent a special challenge in identifying causality through association or linkage studies. Although there is no simple solution to this challenge, improved burden-of-mutation studies could also assist the identification of modifier genes. For example, subjects all having a predicted-deleterious mutation in gene X could be separated into cases and controls on the basis of a gene-X associated trait. Modifier genes could then be identified by carrying out genome-wide association (using either common variants or burden-of-mutation analysis of rare variants). Thus, improved deleteriousness predictions could benefit the study of complex disease.

Pathogenic mutation identification by complementation assays in model organisms has its limits. Such assays are currently only available for a small minority of human genes, not all functions of a pleiotropic human gene may be needed to achieve full complementation, and such assays are unlikely to reveal gain-of-function mutations. However, coupled with rapid advances in sequencing and model organism genetics (Rine 2014), our results support the use of surrogate genetics to identify and characterize pathogenic variants.

## Methods

### Defining the test space of complementation assays

To systematically evaluate yeast/human cross-species complementation in the context of human disease, the test space was defined as follows: first, all yeast/human orthologous pairs were extracted from the InParanoid database (http://inparanoid.sbc.su.se/cgi-bin/index.cgi) (Ostlund et al. 2010). Pairs not meeting all of the following four criteria were eliminated: (1) human genes must have a disease-associated variant identified in either OMIM or HGMD; (2) human genes must have had an open reading frame (ORF­) clone available in the Human ORFeome v8.1 library (Yang et al. 2011); (3) yeast genes must be essential (Winzeler et al. 1999; Giaever et al. 2002); and (4) yeast genes must have an available temperature-sensitive (ts) mutant (Ben-Aroya et al. 2008; Li et al. 2011). In addition to published ts strains, 36 additional strains were obtained through mutagenesis and screening (G Tan, BJ Andrews, and C Boone, unpubl.).

### Construction of the *Saccharomyces cerevisiae* expression plasmid pHYC-NatMX-ORF/GFP

A Gateway cloning destination vector was constructed from the pHiDest-DB (CEN/ARS-based, *ADH1* promoter, and *LEU2* marker). Two-step modification of the original pHiDest-DB resulted in two Gateway-compatible destination vectors with different selection markers. First, the entire GAL4 DNA-binding domain was deleted from the pHiDest-DB resulting in pHYCDest-LEU2 (for use in strains for which the ts allele is linked to NatMX). Next, pHYCDest-natMX (for use in strains for which the ts allele is linked to KanMX) was constructed by replacing LEU2 with natMX in pHYC-LEU2. These modifications were achieved by separate PCR amplification of the pHiDest-DB backbone and natMX4 cassette followed by homologous recombination in yeast. Sequences of primers used are listed in Supplemental Table S5.

The wild-type or mutated disease-associated ORFs and the *GFP* gene were transferred into the pHYCDest by Gateway LR reactions followed by transformation into NEB5α competent *E. coli* cells (New England Biolabs) and selection for ampicillin resistance. After confirmation of ORF identity and expected mutations by Sanger sequencing, plasmids expressing wild-type ORFs, mutated ORFs, and GFP were further transformed into the corresponding yeast ts or haploid-convertible heterozygous diploid knockout mutants.

### Literature curation

Starting from a list of 205 essential and 741 nonessential yeast genes, human orthologs were identified, and curators (R.O., J.H., and C.L.T.) reviewed the published literature for evidence of functional complementation. Complementation of the mutant yeast gene was assessed as either partial or complete restoration of the wild-type yeast phenotype by the human ortholog. The literature was examined as follows: data from studies prior to 2009 were downloaded from the P-POD database (http://ppod.princeton.edu/; version 4, Dec. 15, 2009) (Heinicke et al. 2007) and reviewed; gene identifiers of orthologs were updated as necessary. Papers containing complementation studies not cited in PPOD were obtained through review of the file gene_literature.20140118.tab.gz from the *Saccharomyces* Genome Database (SGD; http://www.yeastgenome.org) (Cherry et al. 2012). This file was screened for papers published through 2012 that had been tagged by SGD curators with the term “cross-species expression.” In 2013, use of this tag was discontinued by SGD; thus all identified yeast papers published from mid-2012 to February 2014 were manually screened for complementation data. Papers identified as having functional complementation data were carefully read, and the relevant NCBI gene identifiers (http://www.ncbi.nlm.nih.gov/gene/) were assigned. Genetic experiments indicating suppression by downstream (or bypass) mechanisms were not considered complementation experiments. Neither were cases where the human gene impaired growth of the yeast mutant, but a mutated version of the human gene rescued growth of the yeast strain. For the subset of 139 human-yeast orthologous pairs in Supplemental Table S1, curation of the published literature revealed 15 complementation relationships involving 15 unique yeast genes and 15 unique human genes. For all essential and nonessential genes with disease-related human homologs, curation of the published literature revealed 197 human-yeast complementation relationships corresponding to 164 human genes (Supplemental Table S2). Information for additional kinds of complementation experiments was curated in this effort (explicit lack of complementation by human proteins, complementation by nonhuman proteins) and can be found in P-POD and downloaded from the ftp site.

### Yeast spotting assays

For yeast ts mutants transformed with expression vectors, cells were grown to saturation in 96-well cell culture plates at room temperature. Each culture was then adjusted to an OD_600_ of 1.0 and serially diluted to 5^−1^, 5^−2^, 5^−3^, 5^−4^, and 5^−5^. These cultures (5 μL of each) were then spotted on YPD plates supplemented with clonNAT or on SC−LEU plates as appropriate to maintain the plasmid and incubated at either 24°C, 30°C, 32°C, 34°C, 36°C, or 38°C for 2 d. Haploid-convertible heterozygous diploid knockout mutants were transformed with expression vectors and incubated in sporulation medium for 5 d at 25°C with shaking. Each culture was serially diluted and spotted as above onto haploid selection plates (Baryshnikova et al. 2010) supplemented with G418 to select for the yeast gene deletion marker. Plates were imaged after 2 or 3 d depending on the growth. Results were interpreted by comparing the growth difference between the yeast strains expressing human genes and the corresponding control strain expressing the *GFP* gene. Two independent cultures were grown and assayed for each strain.

### Yeast liquid growth assays

Exponential growth rates were measured in YPD + clonNAT or other liquid media at 38°C or 36°C. In a 96-well cell culture plate, approximately 10^5^ cells were inoculated into 100 μL liquid medium and the absorbance at 600 nm was read every 15 min using a Tecan GENios Microplate Reader. Growth was calculated for each culture as the slope of the growth curve during log phase over the first five doubling times after detectable growth. Two independent measurements were performed for each strain.

### Site-directed mutagenesis

Site-directed mutagenesis was performed using the Thermo Scientific Phusion Site-Directed Mutagenesis Kit according to the manufacturer's instructions. The Gateway donor pDONOR223-wt_hORF plasmid was amplified using phosphorylated primers that introduce the desired changes, followed by a 5 min room temperature ligation reaction. The resulting plasmid was then transformed into NEB5α competent *E. coli* cells (New England Biolabs). Sequences for all primers used are listed in Supplemental Table S5.

### Predicting functional effects for missense variants

Two functional-assay scores, a functional complementation by spotting score and a functional complementation by liquid growth time-course score, were generated based on the above-described yeast spotting and growth curve assays, respectively. Four semiquantitative FCS scores were assigned to each variant: 0 (wild-type-like complementation), 0.6 (reduced complementation), 0.8 (severely reduced complementation), and 1 (complete loss of complementation). Numerical values were somewhat arbitrary but chosen in rough correspondence to the PolyPhen2 scale of 0 to 1, with scores greater than 0.5 indicating deleterious variation. FCT scores were also calculated on a 0 to 1 scale as (1–µ_VARIANT_)/(1–µ_GFP_), where µ is the relative growth rate calculated as the growth rate of the ts yeast strain expressing a mutant allele (or *GFP* gene) divided by the same ts yeast strain expressing the corresponding wild-type allele.

As described in Results, the uroporphyrinogen III synthase (*UROS*) gene was scored differently. Four FCS scores were assigned to each *UROS* variant: 0 (wild-type-like complementation), 0.6 (slightly increased complementation), 0.8 (increased complementation), and 1 (greatly increased complementation). FCT scores were calculated as follows: (µ_VARIANT_–1)/(µ_FASTEST_–1), where µ is the relative growth rate calculated as the growth rate of the ts yeast strain expressing a mutant allele or the *GFP* gene divided by the same ts yeast strain expressing the corresponding wild-type allele and µ_FASTEST_ is the relative growth rate of the fastest-growing mutant yeast strain.

### Combining experimental and computational scores to predict pathogenic variants

We examined whether prediction performance could be improved by combining FCS and PolyPhen2 scores. To achieve a common scale, precision-calibrated versions of FCS and PolyPhen2 scores (FCS′ and PolyPhen2′) were generated. To avoid circularity that might result from using the observed results for an allele to calibrate the score for that allele, alleles were randomly divided into 10 sets, carrying out calibration for each held-out set in turn, using the remaining nine sets as training data. To calculate the calibrated score for each allele, the corresponding FCS (or PolyPhen2) score was used as a cutoff value to calculate precision within the FCS (or PolyPhen2) training data. This precision value was then taken to be the calibrated FCS′ (or PolyPhen2′) score. Precision and recall performance was then evaluated for seven methods of combining the two scores: minimum, maximum, mean, and four alternative weighted means w1 (0.9 × FCS′ + 0.1 × PolyPhen2′), w2 (0.8 × FCS′ + 0.2 × PolyPhen2′), w3 (0.7 × FCS′ + 0.3 × PolyPhen2′), and w4 (0.6 × FCS′ + 0.4 × PolyPhen2′) (Supplemental Fig. S9C,D). The results show that combining FCS and PolyPhen2 scores can improve the performance in the high precision/low recall region and permit ranking among the many ties that would result from use of the semiquantitative FCS or FCS′ scores.

### Causality of disease-associated mutations

Based on the HGMD mutation annotation, mutations annotated as “DM” (disease-causing mutations) were considered to be causal mutations, while mutations annotated as “DM?” (likely disease-causing mutations), “DP” (disease-associated polymorphisms), “DFP” (disease-associated polymorphisms with additional supporting functional evidence), and “FP” (in vitro/laboratory or in vivo functional polymorphisms) were considered to be noncausal mutations.

### Structural analysis

Protein structure files were downloaded from the Protein Data Bank (Berman et al. 2003). For proteins with more than one solved crystal structure, we chose the structure with the best nominal resolution. Protein structure ‘cartoons’ were colored according to secondary structures. Protein functional sites were retrieved from the Catalytic Site Atlas (Porter et al. 2004) and the PhosphoSite database (Hornbeck et al. 2004). Alpha-carbon pairwise distances between mutated residues and functional sites were calculated using the PyMol software (http://www.pymol.org). For each structure, mutated residues are shown as pink balls and sticks, and functional sites are shown as cyan balls and sticks.
